# Normalization of heart rate variability with taurine and meldonium complex in post-infarction patients with type 2 diabetes mellitus

**DOI:** 10.25122/jml-2019-0052

**Published:** 2019

**Authors:** Juliia Belikova, Victor Lizogub, Andrii Kuzminets, Iryna Lavrenchuk

**Affiliations:** 1. Department of Internal Medicine No 4, Bogomolets National Medical University, Kyiv, Ukraine; 2. Department of the Therapy, Infectious Disease and Dermatology Postgraduate Education, Bogomolets National Medical University, Kyiv, Ukraine

**Keywords:** postinfarction cardiosclerosis, 2nd type diabetes meldonium, variability of heart rate, taurine, meldonium

## Abstract

The purpose of this study is to scrutiny the Dynamics of heart rate variability (HRV) in patients with PICS with 2nd type DM against the background of Taurine (TN) and meldonium (ME). The results of the investigations prove the decrease of the oxidative stress, which is basis of DACN, under the influence of sulfur-containing amino acid taurine (TN), and meldonium (ME) — a competitive inhibitor of gamma-butyrobetaine hydroxylase. Biochemical mechanisms of synergistic action of ME and TN are also described. The results of the studies of 98 patients with PICS and concomitant 2nd type diabetes mellitus were analyzed. They were distributed by simple randomization method into two groups, comparable according to age and sex: the main group (MG) (n = 68): and group of comparison (GoC) (n = 30). HRV was evaluated twice daily at the Cardiosense HMEGG system: at baseline and after 12 weeks of treatment. For the assessment of HRV the frequency and spectral parameters were used. While evaluating the different methods of treatment, their influence on the range of spectral and time indices of HRV was determined (p = 0.001 by the criterion of Kruskall-Wallis). It was learned that the combined application of ME and TN gives a statistically significant (p <0.01) increase of SDNN, HF at night, pNN — on 50% by day (p <0.01, p <0.001 and p <0.01 respectively), and statistically significant decrease in LF at night, compared to GHG.

## Introduction

Low heart rate variability (HRV) as a manifestation of diabetic autonomic cardiac neuropathy (DACN) has a high correlation with the risk of sudden coronary death. [1–4]. Sympathicovagal imbalance (SVI) is especially pronounced in patients with postinfarction cardiosclerosis (PICS) and is a prerequisite for the emergence of life-threatening high-grade arrhythmias [5–11]. The latter cause deaths of almost half of patients with 2nd type diabetes mellitus (DM 2), and this indicator has a tendency to increase [12–14].

All antiarrhythmic drugs classified by Voughan-Williams have a number of side effects and contraindications, and a significant amount is characterized by a proarrhythmogenic effect [15–18].

A promising direction may be the use of drugs that affect the energy metabolism of the myocardium (M).

Ability of metabolic therapy (MT) to increase the stability of the nervous system (NS) to adverse factors of different origin, activate plastic processes in the central nervous system (CNS), normalize the energy status of nerve cells is proven. In addition, the peculiar feature of MT is the practical absence of complications and side effects and the possibility of their combination with other drugs.

Important predictors of endogenous response to the treatment are amino acids (AMAs). [19–24]. The latter are the regulators of enzymes and substrates activity of a number of Biochemical reactions, which are especially relevant in the case of absence of positive Dynamics during the use of MT, when some changes in treatment policy are necessary .

TN is a sulfur-containing amino acid, which remains in free form, unlike other amino acids that can be used as substrates for the formation of other proteins. It is proved that TH is one of the main regulatory substances that restore the neurodynamic and sympathetic-parasympathetic balance (SPB). [25,26]. In the experimental conditions, morphofunctional changes in the structures of the central nervous system, which are responsible for the visceral sphere and SPB, against the background of introduction and acceleration of adaptation processes under the influence of TN during the early post-stress period, were revealed. The involvement of TN in the decrease of the operating system, which is the basis of the DACN, were proved [27].

ME is a competitive inhibitor of gamma-butyrobateine hydroxylase, which inhibits intracellular synthesis of carnitine. The latter is responsible for the transport of fatty acids (FA) through the membrane of mitochondrias. The results of the investigations confirm the tonic effect of ME on the CNS and the vividly expressed antiarrhythmic activity [30–32].

It was proved that inhibition of the stearoyl-CoA desaturase enzyme (SCD-1) suppresses oxidation of the FAs and increases the tissue’s sensitivity to insulin. And this very enzyme is a connecting link between AMAs and FA. Expression of this enzyme is proved in the relation to TN. The synergistic effect of ME and TN can be explained by above mentioned Biochemical interactions [33].

Consequently, the multiple use of ME and TN in patients with PICS and 2nd type diabetes is not described in previous studies, but a significant amount of such the data on their isolated effects on HRV and mutual action potentiation has been accumulated.

Purpose: to learn the Dynamics of HRV indices in patients with PICS and 2nd type DM against the background of the use of taurine (TN) and meldonium (ME).

## Materials and Methods

The results of a survey of 98 patients with PICS and concomitant 2nd type DM were analyzed, which were distributed by simple method of randomization into two groups: main group (MG), (n = 68): 36 women and 32 men, median age of patients — 65.5 years (IQR (interquartile range) — 61-68 years), and the group of comparison (GH) (n = 30): 17 women and 13 men, median age - 64.7 years (IQR — 64-68 years). The investigations involved patients with PICS and associated 2nd type DM who were informed and agreed to the experiment. The control group (CG) was represented by 30 persons, age and gender were in comparison with PICS and 2nd type DM patients.

Criteria for exclusion from the study: chronic heart failure IIB-III stage according to Strazhesko-Vasilenko classification, acute coronary syndrome during the last 12 months, congenital and acquired heart defects, complete blockade of LBBB, implanted pacemaker, AV-blocks II-III of II-III stage, atrial fibrillation, autoimmune diseases, malignant oncological diseases, expressed renal, hepatic, respiratory failures, endocrine diseases (except 2nd type DM).

The general clinical characteristics of patients is shown in the Table 1.

**Table 1:** The general characteristics of examined patients (Me (IQR))

Index	Examined patients, n= 98
Age	64 (60-69)
Sex: male/female, n ,%	50 (51.0%)/48(48.97%)
Age of DM, years	7.69 (7.45-8.12)
Age of, years	6.09 (5.35-8.02)
Body Mass Index, kg/ᴍ^2^	31.72 (27.8-39.11)
Accompanied cardiac unsufficiency (CН I), n, %	98 (100.0 %)
Accompanied arterial hypertension, n (%)	98 (100.0 %)
Homa index	6.02 (5.11-6.16)
Official SBP, ᴍillimeters of mercury	144.2 (126.7-154.5)
Official DBP, ᴍillimeters of mercury	87.45 (86.7-88.37)
С-reactive protein, ᴍg/L	3.74 (3.12-5.11)
Glycosylated hemoglobin, %	7.92 (6.16-8.3)

Patients from main group received basic treatment (BT), which included an ACE inhibitor, beta-adrenoblocker, statin, antiaggregant, oral hypoglycemic therapy, and additionally 1 capsule 500 mg 2 times per day of ME and additionally 1 capsule 400 mg of ME 3 times per day and 1 capsule 400 mg of TN. 3 times per day. Patients from GoC received only BT PICS and 2nd type DM drugs. In order to study the complex and isolated effects of TN and ME on the frequency of ADR, MG was divided into 3 subgroups according to the additional BT treatment BT. The 1st subgroup (GoC 1st) included 22 patients, who received TN, 2nd subgroup (GoC 2nd) — 23 patients, receiving ME, 3rd subgroup (GH 3) — 23 patients, who received combined ME and TN in addition to BT.

HRV was evaluated twice daily at the Cardiosense HMEGG system: at baseline and after 12 weeks of treatment. For the assessment of HRV was used the frequency and spectral parameters. We studied the following frequency characteristics of HRV: SDNN(MS) – standard deviation of all intervals R-R; RMSSD (MS) – the square root of the mean sum of squares of differences between adjacent intervals R-R; pNN50 (%)- the number of pairs of neighboring intervals R-R ,which differ more than 50 MS in the entire recording. Among the following spectral indices: TP – Total power (MS2) total power of HRV spectrum, LF (MS2) – slow wave (low frequency) part of the spectrum in the frequency range from 0.04 to 0.15 Hz, VLF (MS2) very low frequency part of the spectrum in the range of 0.0033–0.04 Hz, VLF (MS2) very low frequency part of the spectrum in the range of 0.0033-0.04 Hz, UVLF (MS2) ultra low frequency component with the frequency of the wave to 0.0033 Hz, HF (MS2) – high frequency component in the frequency range from 0.15 to 0.5 Hz, the ratio LF/HF sympatho-wagusnye of balance. Used software allowed to carry out a dynamic assessment of frequency and spectral indices of HRV in the daytime and during sleep. In order to exclude the influence of defects of record, artifacts, noise in the analysis of HRV parameters was carried out a thorough analysis of all the unidentified QRS complexes and artifacts. In measuring and evaluating the results of the ECG EC, the recommendations of the Task Force of the European Society of Cardiology and the North American Society of Cardiac Stimulation and Electrophysiology were followed [34]. Statistical data analysis was carried out using SPSS, MedStat, EZR packages. The verification of normal distribution of parameters was evaluated by using the Shapiro-Wilk W-test. The comparison of uninterrupted values was performed by using the U-criterion of Mann-Whitney U-test, as their distribution varied from a normal one. Quantitative data are represented as Me (IQR), where Me means the median, IQR means the interquartile range (the first and third quartiles). The Dann’s test was used for multiple comparisons.

## Discussion and Results

Thus, according to the HRV analysis, there was a significant decrease of one of the most sensitive indicators of HRV-SDNN compared with CG patients with PICS and 2nd type DM (p <0.05). The latter characterizes the total effect of vegetative regulation of blood circulation. At the same time, it was monitored a significant (p <0.05) decrease in RMSSD compared to GOC in patients with PICS and DM, indicating a decrease in the activity of the parasympathetic nervous system (PNS).

Relative (p <0.05) decrease in mRR in patients shows a relative increase in the effects of the sympathetic nervous system (SNA) during the day. This is also confirmed by the probable (p <0.05) decrease in the circadian index in these patients compared with GOC patients. Rigidity of HRV appeared in a peculiar way in patients with PICS and DM 2 as a result of weakening of vagal influences. The latter fact indicates the presence of autonomic myocardial denervation (Table 2).

**Table 2:** Time rates of HRV in patients with PICS and 2nd type DM against the background of taking taurine and meldonium dihydrate (Me (IQR))

Index	Period of day	Main group patients (n=98)						Group of Comparison n=30		Control group n=30
		1st Subgroup n=22		2nd Subgroup n= 23		3rd Subgroup n= 23				
		1	2	1	2	1	2	1	2	
mRR	Day	860.61 (845.3-871.22)	887.4(865.2-891.1)	854.3 (842.1-861)	873.22 (868.1-881.3)	864.3(859-867)	910.21 (907.13-914)	843.2 (831.1-856)	846.2 (841.1-852.1)	899.3 (871.1-901.1)
	Night	951.1 (931.1-972.2) #	961.1 (915.1-971.1)	873.2 (861-881.1) #	961.4 (951.1-971.2)	957.2 (951.1-959) #	997.2(992-998)	953,1 (952.6-961) #	954.3 (952.2-961)	915.43 (913.4-931)
SDNN	Day	36.41 (35.21-37.2) #	37.45 (32.65-39.1)	36.33 (35.77-37.9) #	38.11 (37.87-41.1)	35.12 934.67-37.2) #	41.11 (40.54-42.3) ^	35.34 934.32-36.78) #	35 .58 (32.12-38.91)	47.14 944.31-51.2)
	Night	41.13 (40.36-42.16) #	42.2 (41.65-43.1)	40.2 940.1-41) #	41.3 940.34-42) #	42.4 (41.5-45) #	45.03 (44.2-44) * ^	42.1 (41.1-43) #	43.2 (42.4-44)	59.11 (56.8-61)
RMSSD	Day	19.7 (18.3-21)	21.3 920.1-22)	24.01 923-25.2)	25.1 (24.4-27)	20 .3 919.9-24)	23.1 (22.1-25) *	20.2 (18.9-22)	21.2 (19.9-21)	24.1 (23.2-25)
	Night	24.1 (23.1-26)	26.2 (23-27.2)	31.1 (28.2-34)	34.2 (33.1-35)	26.2 (25.3-27)	33.1 (31.2-34) *	28.2 923-29.2)	35.4 (34.1-37)	31.2 (30.3-34)
pNN 50, %	Day	2.5 (1.97-2.7)	2.7 (2.34-2.8)	2.3(2.21-2.6)	2.4(2.23-2.7)	2.8 92.54-2.9)	4.1 (3.97-4.13) * ^	2.9 (2.87-3.12)	3.3(3.1-3.7) ^	6.3 (6.1-6.21)
	Night	4.5 (4.41-4.7)	4.7(4.6-4.81)	6.1 95.93-6.1)	6.2 (6.13-6.23)	4.9 (4.7-5.1)	6.12 (6.1-6.17)^	5.1(4.97-5.4)	5.1(5.0-5.15)	8.1 (8.1-8.65)
Circadian index,	-----	1.2 (1.17-1.3) #	1.25(1.24-1.27)	1.9 (1.8-2.1) #	1.24 (1.17-1.26)	1.2 (1.14-1.25) #	1.27 (1.24-1.3)	1.2 (1.15-1.28) #	1.22 (1.17-1.29)	1.34 (1.31-1.4)

Notes: before 1st-treatment, after 12 weeks of treatment, # - p <0,05 ## - p <0,001 compared to GOC * - p <0,01 ** - p <0,001 - compared with the indicators up to 12 weeks of treatment, ^ - p <0,01 ^^ - p <0,001 - compared with GH after 12 weeks of treatment

While the spectral analysis of HRV conducting, a significant decrease in the total spectral power (TP, p <0.001) was found in patients with PICS with concomitant 2nd type DM, as compared with those of GOC patients. It is characterized by a decrease in ТР in the daytime due mainly to very slow (VLF, p <0.001) and slow (LF, p <0.001) waves, which determine the activity of the SNA and humoral and metabolic effects. At night, changes in ТР were due to a decrease in power and fast waves (HF, p <0.001), which characterizes the activity of PNS. The growth of the LF / HF index in the examined patients compared with the GOC patients indicates a relative prevalence of the SNA in patients with PICS and 2nd type DM. It can be the result of a decrease in the sensitivity of the sinus node to the nerve regulation due to the steady activation of the SNA.

In the study of the effectiveness of the multiple treatment of ME and TN, a significant (p <0.01) increase in SDNN was observed against the background of the use of ME and TN in the night period in all subgroups of MG, especially expressed in SG3. At the beginning of investigations, before the appointment of ME and TN, all subgroups of MG and GOC were comparable according to the values of time and spectral indices of the HVR (STI HRV). Thus, the multiple treatment of ME and TH contributed to reducing the tension in the functioning of the SNA. Normalization of RMSSD was noted, and pNN increase on 50% by day. The most vivid in comparison with the initial stage of the study was the change in SG 3.

While analyzing the spectral indices of HRV, it was learned that the multiple treatment of ME and TH contributed to the decrease of VI. These results were reflected in a significant (p <0.01) decrease of LF and increase of HF at night, vividly expressed in patients with SG3 (Table 3).

**Table 3:** Spectral indices of HRV in patients with PICS and 2nd type DM against the background of receiving taurine and meldonium dihydrate (Me (IQR))

Index	Main group, n= 68							Group of Comparison n= 30			COG n= 30		
	1st Subgroup n=22			2nd Subgroup n=23		3rd Subgroup n=23							
	Day		Night	Day	Night	Day	Night		Day	Night		Day	Night
TP, mc2	1	1523.54 (1487.6-1525) ##	1870.81 (1756.1-1223) ##	1531.1 (1518-1541) ##	1910.1 (1907-1913) ##	1528.2 (1514-1531 ##)	1925.1 (1914-1917.1 ##)		1532.2 (1527-1534) ##	1911.12 (1905-1923) ##		3421.15 (3420-3423)	3120.11 93101-3123.5)
	2	1630.13 (1625-1634)	2010.1 92007-2012.1)	1634.3 (1631-1643)	1997.2 (1993-1999)	1970.2 (1965-1974)	2350.2 (2346-2356)		1643.4 (1634-1651)	1987.1 (1983-1991)			
VLF, mc2	1	705 ##	951.52 (881-1015.67)	707.5 (705.3-709.4) ##	934.3 (928.1-941.2)	710.5 (707.9-711.2) ##	921.2 (918.1-923.2)		707.3 (705.1-709.7) ##	924.3 (921.4-926.7)		1110.3 (1101-115.5)	1311.2 (1223-1345.2)
	2	711.2 (707-714)	972.2 (968-974)	717.79 (715-719)	961.2 996=57-964)	753.2 (751-755)	1017.2 (1015-1019)		709.2 9707.3-711)	943.2 9941-955)			
ULF, mc2	1	552.68 (478-565.6)	407.31 (398.61-415.8)	557.2 (551-561)	410.12 (404-412)	553.1 (551-557)	410.2 (407-412)		543.2 (541-551)	411.2 (407-414)		984.13 (981-991)	557.12 (551-559)
	2	572.2 (571-575)	412.3 (411-414)	565.1 (562-571)	421.2 (419.1-424)	640.2 (638-641)	450.2 (447-454)		545.1 (537-545)	412.2 (411-415)			
HF, mc2	1	110.12 (98.61-115.63)	201.43 ## (178.6-215.65)	112.2 (1.1-113)	205.3 (201-207) ##	112.1 (11.3-113)	203.1 (202-205) ##		110.2 (108-111)	207.1 (25-2111.3) ## ^^		365.43 (363-341.2)	515.3 (513-517)
	2	134.2 (112-136)	223.4 (221-225.1)	143.1 (137.1-142)	251 (243-254.7)	190.7 (187-201)	291.3 (296-302) **		143.6 (141-147)	214.5 (212-216)			
LF, mc2	1	220.76 ## (198.61-235.6)	421.49 ## (376.6-515.03)	223.12 (221-225.2) ##	421.2 (419-423.2) ##	231.2 (230-234.2) ##	430.3 (427-432.5) ##		223.3 (221-225) ##	427.2 (424-431)##		650.12 (645-661.2)	702.13 (701-705.5)
	2	241(237-251)	441 (367-487)	236 (224-254.1)	441.3(427.6-451.2)	291.3 (278.3-301.1)	460.2 (457.1-462.3)		236.4 (234.5-239.3)	429.1(418.7-432.2)			
LF/HF	1	2.6 (2.1-2.7) ##	2.4 (2.1-2.8) ##	2.7(2.2-2.9) ##	2.5(2.1-2.9) ##	2.8 (2-3.1)##	2.5(1.9-3) ##		2.9(1.5-3) ##	2.4 (2-2.6) ##		2.1 (1.9-2.5)	1.72 (1.3-1.9)
	2	1.9 (1.8-2.1)	2.2 (1.8-2.5)	2.3 (1.9-2.1)	2.3 (1.5-2.5)	1.6(1.4-1.9)	1.5 (1.3-1.7)		2.6(2.1-2.8)	2.3 (2.1-2.8)			

Notes: before 1st-treatment, after 12 weeks of treatment, * - p <0.01, ** - p <0.001 - compared with the indices before treatment, ## - p <0.001 compared with GOC, ^ - p <0.01 ^^ - p <0,001 -compared with SG after 12 weeks of treatment.

While evaluating the different methods of treatment, their effect on the STI HRV indices was proved (p = 0.001 by Kruskall-Wallis test). It was found that the multiple treatment of ME and TN gives statistically significantly higher growth of SDNN, HF at night, pNN – on 50% by day (p <0.01, p <0.001 and p <0.01 respectively), and a statistically significant decrease in LF at night, compared with SG.

Statistically significant differences in growth of SDNN, HF at night, pNN – on 50% in the afternoon and LF decrease in night PG1 and PG2 between themselves and with GOC were not detected (p> 0.05).

## Conclusions

The violation of autonomic regulation of cardiac activity in patients with PICS and 2nd type DM is characterized by vegetative imbalance and weakening of humoral, metabolic and vagal influences, relative sympathysation, decreased PNS activity. While evaluating the influence of various methods of treatment on the value of STI HRV It was learned, that the multiple treatment of TN and ME in addition to BT during 12 weeks in patients with PICS and 2nd type DM positively affects the STI HRV and also normalizes the vegetative balance by reducing the tension in the functioning of the SNA and activating parasympathetic rhythm regulation. Statistically significant differences STI HRV changes while comparing separate 12-week appointment of ME in addition to BT and a separate appointment of TN in addition to BT within 12 weeks, with each other and with BT treatment was not detected (p> 0.05).

## Conflict of Interest

The authors confirm that there are no conflicts of interest
